# Survivors

**Published:** 1891-11-21

**Authors:** 


### Passing Topics.

### SURVIVORS.

In all likelihood most people who have read the correspondence in the papers between Mr. Ribton Turner and the Resident Medical Officer of St. Thomas's, will agree that the matter has not been disposed of very satisfactorily. In the interests of the hospitals it is desirable that a charge publicly made against the administration of any one of them, especially when made by a responsible accuser, should be promptly and adequately met. In the instance under notice, we scarcely think the verdict will be that the hospital authorities have taken the course best calculated to satisfy legitimate anxiety and to allay natural apprehensions. As we gather from the complaint, a patient was admitted to undergo a certain operation. The authorities were warned that he had a weak heart, and in their discretion they administered chloroform to him, "with the result that in less than three minutes he ceased to breathe." It is alleged further that the methods of resuscitation which might have been employed were neglected, and when the inquest was held, the feelings of the friends were hurt by the absence of those who ought to have been present, involving a postponement of the enquiry, and that the "flippant treatment" accorded the matter has added much to the pain suffered by the family. The answer publicly given to this is at least insufficient, and is of a nature ill-designed to allay irritation. Room is found to interpolate an expression of sympathy, but the gist of the reply is—if you are not content, you are at liberty to address the Treasurer.

It is the fault of the institution, or rather of its representative, if the public fail to do it justice. For our own part, knowing what we do of the care bestowed in our hospitals, and certainly not less in St. Thomas's than elsewhere, upon the humblest patient, and the scrupulous attention paid to the smallest details of surgical work, we will not for a moment believe, in the absence of positive proof, that anything was wanting in respect of the operation itself. But what we fear may be truthfully asserted as a general charge, is that when the resources of science are exhausted, and the end has come, there is now and again too little evidence of consideration for the natural feelings of the friends. Here was a case where the concomitant circumstances were especially distressing, and if the character of our hospitals for humanity is to be maintained unimpaired, we must not exclude them from our reckoning. It is not enough to deal with the patient in the abstract, however tenderly and conscientiously. To attempt it only lands us in the position that when the patient is dead, there the matter ends, and that is precisely what it does not do—at least, so far as the friends are concerned. Medical training, it must be admitted, does not encourage a sense of excessive respect for the body when life is extinct. To a layman, unless he be unnaturally callous and ghoul-like, or unless circumstances have made him unusually familiar, there always comes a feeling of awe in the presence of death, and the last offices are rendered in a reverential spirit. The scientific mind is often purged of weaknesses of this description, and without any intentional want of feeling, the sensibilities of the survivors are overlooked. We see in what has occurred in the present case a fresh indication of the importance of upholding the benevolent, that is the lay, side of our hospital work intact. Medical science is essentially a philanthropic agent. It aims at benign and far-reaching results, and for that reason it cannot always be trusted to do present justice to its own unimpeachable principles.

Agricola.

